# Cognitive and Psychological Reactions of the General Population Three Months After the 2011 Tohoku Earthquake and Tsunami

**DOI:** 10.1371/journal.pone.0031014

**Published:** 2012-02-08

**Authors:** Yasushi Kyutoku, Ryoko Tada, Takahiko Umeyama, Kenji Harada, Senichiro Kikuchi, Eiju Watanabe, Angela Liegey-Dougall, Ippeita Dan

**Affiliations:** 1 Functional Brain Science Laboratory, Center for Development of Advanced Medical Technology, Jichi Medical University, Tochigi, Japan; 2 Department of Marketing and Research, Cross Marketing Inc. Tokyo, Japan; 3 Department of Psychiatry, Jichi Medical University, Tochigi, Japan; 4 Department of Neurosurgery, Jichi Medical University, Tochigi, Japan; 5 Department of Psychology, College of Science, University of Texas, Arlington, Texas, United States of America; Chiba University Center for Forensic Mental Health, Japan

## Abstract

**Background:**

The largest earthquake on record in Japan (magnitude 9.0) occurred on March 11, 2011, and the subsequent tsunami devastated the Pacific coast of Northern Japan. These further triggered the Fukushima I nuclear power plant accidents. Such a hugely complex disaster inevitably has negative psychological effects on general populations as well as on the direct victims. While previous disaster studies enrolled descriptive approaches focusing on direct victims, the structure of the psychological adjustment process of people from the general population has remained uncertain. The current study attempted to establish a path model that sufficiently reflects the early psychological adaptation process of the general population to large-scale natural disasters.

**Methods and Findings:**

Participants from the primary disaster area (*n* = 1083) and other areas (*n* = 2372) voluntarily participated in an online questionnaire study. By constructing path models using a structural equation model procedure (SEM), we examined the structural relationship among psychological constructs known related to disasters. As post-traumatic stress symptoms (PTS) were significantly more present in people in the primarily affected area than in those in secondary- or non-affected areas, the path models were constructed for the primary victims. The parsimoniously depicted model with the best fit was achieved for the psychological-adjustment centered model with quality of life (QoL) as a final outcome.

**Conclusion:**

The paths to QoL via negative routes (from negative cognitive appraisal, PTS, and general stress) were dominant, suggesting the importance of clinical intervention for reducing negative cognitive appraisal, and for caring for general stress and PTS to maintain QoL at an early stage of psychological adaptation to a disaster. The model also depicted the presence of a positive route where positive cognitive appraisal facilitates post-traumatic growth (PTG) to achieve a higher QoL, suggesting the potential importance of positive psychological preventive care for unexpected natural disasters.

## Introduction

The largest earthquake on record in Japan with a magnitude 9.0 on the Richter scale took place on March 11^th^, 2011 [1], and a subsequent tsunami devastated the Pacific coast of Northern Japan . The earthquake and tsunami further triggered the Fukushima I nuclear power plant accidents with three reactors melting down: the largest nuclear accident since the 1986 Chernobyl disaster. Such a huge, complex disaster inevitably has negative psychological effects on general populations as well as on immediate victims, but since such disasters hit unexpectedly, previous disaster studies have tended to rely on a descriptive approach focusing on immediate disaster victims. This has precluded holistic analyses of the psychological adjustment process of general populations effected by disaster. Here, we performed the first comprehensive study on the psychological adjustment to a major disaster in a general population as well as in immediate disaster victims, and, using a path model, we clarified the structural relationships among the process of psychological adjustment following cognitive appraisal of the disaster, mental problems, and quality of life (QoL). While the disaster had a considerable negative mental impact reflected as general and post-traumatic stresses on the general population, the promising sign of a favorable effect post-traumatic growth (PTG) was also visible.

The unexpected, unpredictable, sudden, and massive nature of such a disaster would result in negative psychological changes in a goodly portion of people beyond the immediate victims, and a substantial number of them would suffer from drastic changes for a prolonged period of time [2]. Some of the most prevalent negative psychological changes include increases in acute stress disorder (ASD) and post-traumatic stress disorder (PTSD) [3] [4]. ASD may be transient, lasting about four weeks, and sometimes develops into PTSD and other distressing symptoms including re-experience, avoidance, numbness, and hyperarousal, which last at least a month. Diagnostic criteria for ASD and PTSD based on DSM-IV-TR [5] [6] include 1) intrusion/flashback, 2) avoidance of traumatic stimuli, and 3) hyperarousal. Prevalence of PTSD ranges from 3 to 45.5% [7] [8] [9] [10] [11] [12] of the people affected by disasters. Although cut-off scores are important clinically, it has been reported that there are no significant differences in psychological comorbidities among people above threshold, sub-threshold, and without abnormality [13]. Therefore, to include varying degrees of post-traumatic stress symptoms (PTS) as a continuous measure, rather than as cut-off scores, would be more informative.

In addition to a stress for a specific incident exemplified as PTS, general stress, which is defined as subjective perception of the distressing state of having little control over a situation, has been reported to concurrently increase among disaster victims [2] [14] [15]. For instance, association between PTS and general stress has been indicated: after exposure to the September 11^th^, 2001 terrorist attacks in New York City, later PTSD was associated with the existence of other stressors [16]. PTS and general stress together would exert further damage to mental health, presumably leading to general anxiety and major depression [15]. It should be noted that although PTS is a kind of anxiety symptom, there is a clear distinction between the two: PTS is caused by a specific event while anxiety is fear of a general event which has not happened. This would be especially so for the huge disaster affecting most of the population of Japan.

**Figure 1:**
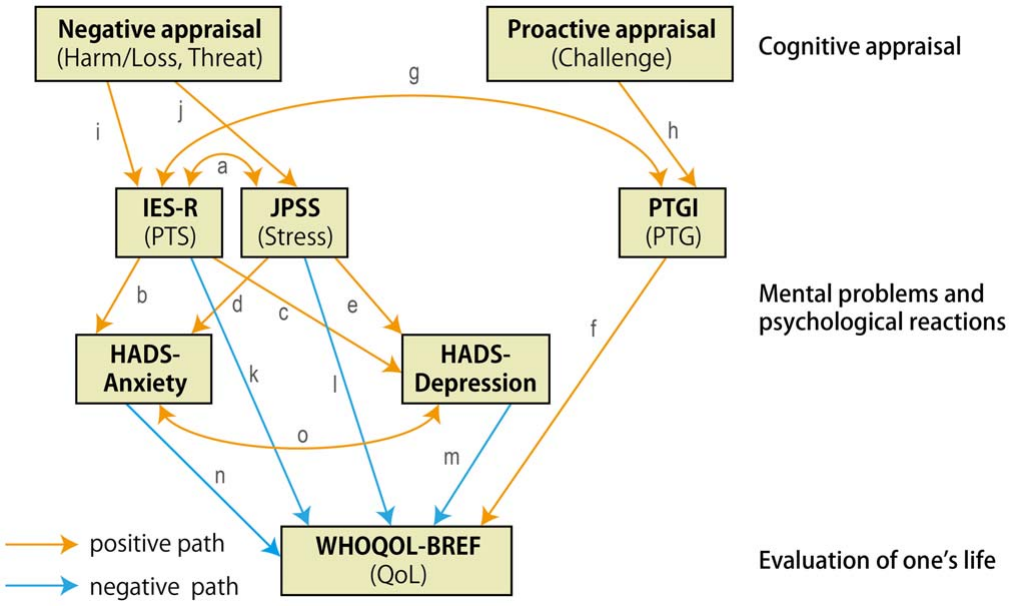
Hypothesized Model 1 with QoL as a final outcome. One-headed arrows indicate the direction of hypothetical regression. Two-headed arrows indicate hypothetical correlation, which are actually among error components of corresponding observed variables, but the error components were omitted for the sake of simplicity. Letters (a to n) indicate hypothetical paths as described in the main text.

**Figure 2:**
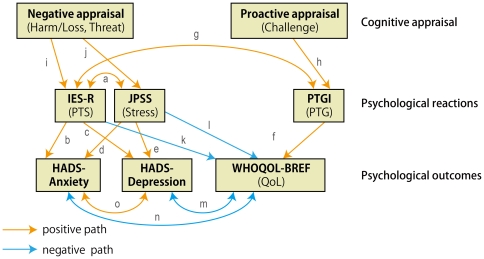
Hypothesized Model 2 with QoL and mental problems as final outcomes. One-headed arrows indicate the direction of hypothetical regression. Two-headed arrows indicate hypothetical correlation, with error components being omitted for simplicity. Letters (a to n) indicate hypothetical paths as described in the main text.

Hypothesis 1 (H1) (Path a in the hypothesis models in Figures 1 and 2). There is a positive association between PTS and general stress.
H2 (Path b, Figures 1 and 2). PTS positively accounts for anxiety.
H3 (Path c, Figures 1 and 2). PTS positively accounts for depression.
H4 (Path d, Figures 1 and 2). General stress positively accounts for anxiety.
H5 (Path e, Figures 1 and 2). General stress positively accounts for depression.

Not only negative psychological changes, but also positive psychological changes such as post-traumatic growth (PTG) appear as a consequence of the aftermath of disasters [17] [18] [19] [20] [21] [22]. PTG is defined as post-trauma improvements in relationships with others, personal strength, spirituality, appreciation of life, and identification of new possibilities in one's life. Moreover, PTG has been reported to account for other psychological outcomes such as improved quality of life (QoL) and to alleviate depression [23]. PTG, an important process of psychological adaptation, is not independent of or negatively associated with PTS, but rather should have a positive correlation with PTS, as PTG is theorized to occur only with the presence of the perception of trauma [17]. Indeed, in a study of the Sichuan earthquake victims, PTS was shown as the strongest correlate of PTG together with a higher degree of earthquake-related exposure [24] .

H6 (Path f, Figures 1 and 2). PTG positively accounts for QoL.
H7 (Path g, Figures 1 and 2). There is a positive correlation between PTG and PTS.

Cognitive appraisal was theorized to precede trauma-related psychological changes (PTS, general stress, and PTG) according to the transaction model of stress. Cognitive appraisal refers to the way individuals evaluate the relationships with their environment, and further to the significance of these relationships to their wellbeing [25]. Some studies dealing with chronic pain revealed that negative cognitive appraisal of pain showed an adversary effect on coping and the overall wellbeing of patients [26] [27] [28]. Specifically, negative appraisals of pain and its consequences tend to lead patients to exert passive coping styles, often resulting in decreased daily activity levels, and further resulting in functional incapacity. On the other hand, there is also the challenge appraisal of pain, referring to the potential for growth, mastery, or gain associated with pain [29]. Specifically, challenge appraisals may lead patients to utilize active coping to adjust well to chronic pain [30]. Regarding PTS, its association has been reported with appraisals of a traumatic event [31], of self [5], and of self-blame for a traumatic event. These reports suggest that negative and challenge cognitive appraisals could be key to elucidating psychological changes after a traumatic event.

H8 (Path h, Figures 1 and 2). Challenge appraisal positively accounts for PTG.
H9 (Path i, Figures 1 and 2). Negative appraisals (harm/loss and threat) positively account for PTS.
H10 (Path j, Figures 1 and 2). Negative appraisals (harm/loss and threat) positively account for general stress.

Subsequent to cognitive appraisal and psychological adjustment (PTS, PTG, and general stress), reduced QoL and mental problems such as depression and anxiety are some of the most frequently reported outcomes [32] [33] [34] [35] [36]. For instance, Morrill reported a positive association between PTS and depression in 161 breast cancer patients [37]. There was a negative association between PTS and QoL, and a positive association between PTG and QoL. Also, there was a positive association between PTS and depression. In addition, there was an interaction between PTS and PTG on QoL and depression (when PTG was lower, PTS was more negatively associated with QoL and more positively associated with depression. As for the association between general stress and QoL, Kocalevent et al. examined the association between stress and QoL in the light of transactional theory of stress in a 2552-person community sample, and they reported moderate to large negative correlations between stress level and QoL [38].

H11 (Path k, Figures 1 and 2). PTS negatively accounts for QoL.
H12 (Path l, Figures 1 and 2). General stress negatively account for QoL.

Although QoL, depression, and anxiety were commonly used as outcomes of psychological path models based on review [39], relationships among them have been reported differently depending on the previous findings. Guan examined the relative impact of various mental problems on QoL, and reported a negative association between mental problems (such as PTS, depression, and anxiety) and QoLs in a 640-person community sample in two U.S. counties [35]. On the other hand, Norberg et al. depicted QoL as a psychological construct with which mental problems are not a criterion, but rather concurrent [32]. Based on their findings, there were negative associations between depression and QoL and negative associations between anxiety (STAI) and QoL. However, there were positive associations between depression and anxiety in participants who sought treatment at a mental clinic.

H13 (Path m, Figures 1 and 2). There is a negative correlation between depression and QoL.
H14 (Path n, Figures 1 and 2). There is a negative correlation between anxiety and QoL.
H15 (Path o, Figures 1 and 2). There is a positive correlation between depression and anxiety.

Other than the psychological constructs mentioned so far, demographic differences such as distance of residence from the epicenter, age, and gender have been repeatedly reported to be associated with PTS after the disaster [40], [41], [42]. Further, social support has been reported to have an alleviation effect on PTS [40].

We aim to combine these findings and present the first comprehensive study, in which two hypothesized models (Model 1: QoL as a final outcome vs. Model 2: QoL and mental problems together as outcomes) are examined using path models reflecting the associations among cognitive appraisal, PTS, PTG, general stress, anxiety, depression, QoL, demographics, and social support after the large-scale natural disaster in Japan, with a central focus on PTS (Figures 1 and 2).

Competing hypothesized models. Depression and anxiety are precedents of QoL (Model 1, Figure 1) or depression and anxiety are concurrent with QoL (Model 2, Figure 2).

The hypothesized path models were examined based on goodness of fit indices using a structural equation modeling (SEM) procedure. It should be noted that the current study not only describes the negative psychological phenomena after the 2011 Tohoku earthquake, tsunami, and nuclear accident, but also provides evidence for positive psychological change after a trauma (PTG) and implication for intervention. Instead of a longitudinal design, the hypothesized models based on preceding studies were examined in order to understand concurrent psychological paths in the early psychological adaptation process to a large-scale disaster at three months after its occurrence.

## Methods

### Participants and procedure

An online survey company (Cross Marketing Inc., Tokyo, Japan) was contracted to collect responses. A stratified random sampling was adopted as described below. The participant pool of the online survey company (N = approximately 1.5 million) was categorized into three groups by their location when the earthquake occurred: (1) the primary disaster area where Japan Meteorological Agency (JMA) seismic intensity was larger than or equal to 6; (2) the secondary disaster area where JMA seismic intensity was larger than or equal to 4 but below 6; and (3) non-affected areas where JMA seismic intensity was below 4. They were further classified by age (five age categories of ten years each from 20s to 60s) and gender, generating a total of thirty quotas. An invitation message to participate in the current study was sent to 43,000 registrants. The number of randomly selected registrants to whom the invitation message was sent was determined based on response rates of past surveys so as to allocate a balanced number of participants to each quota. The invitation expired on the date when at least one thousand participants per group were ensured. Because participation was voluntary, the ratio and profiles of invitees who refused to participate are difficult to estimate.

Consequently, 3,455 Japanese (mean age = 46.5 years old, SD = 12.83 years; 2,010 males and 1,445 females) participated the current study. Specifically, there were (1) 1,083 Japanese in the primary disaster area (mean age = 45.0 years old, SD = 12.4 years; 626 males and 457 females); (2) 1,124 Japanese in the secondary disaster area (mean age = 47.6 years old, SD = 12.6 years; 681 males and 443 females); and (3) 1,248 Japanese in non-affected areas (mean age = 46.7 years old, SD = 13.3 years; 703 males and 545 females).

After agreement, with informed consent, to the terms of this study, participants responded to an impact-of-event scale revised into Japanese (IES-R). Participants, whose IES-R score was above 3 SD (61 out of 88 points), were not allowed to participate in the study in conformance with the institutional ethical committee, were advised to consult the public health care department, and were provided with contact information through indications (redirection to a different page) on their computer screen. The rest of the participants proceeded to the other parts of questionnaire (described in the *Measures* section below). The entire procedure took approximately 50 minutes to complete. Participants were free to quit the survey at any time.

The Internet survey was performed from June 24 to 29, 105 to 110 days after the occurrence of the earthquake.

### Measures

The analyses of the current study employed the responses to the IES-R for the earthquake & tsunami and the nuclear crisis, demographic measures, exposure to the earthquake & tsunami and the nuclear crisis, WHOQOL-26, perceived stress scale, Jichi Medical School Social Support Scale (JMS-SSS), posttraumatic growth inventory (PTGI), cognitive appraisal of health scale (CAHS), and hospital anxiety and depression scale (HADS). PASW-19.0 and AMOS 19.0 were used for the analyses.

### Impact of Event Scale-Revised (IES-R)

The twenty-two item IES-R has been widely used to measure posttraumatic stress symptoms including subscales such as intrusive thoughts and recurrent flashbacks, avoidance, and hyperarousal due to a traumatic source in 5-point Likert-type graded responses (0 = not at all and 5 = extremely). The scale was originally developed and validated by Horowitz, Wilner, & Alvarez [43], and the Japanese translation, back translation, and validation were conducted by Asukai et al. [44]. In the current study, IES-R was used to measure posttraumatic symptoms (1) from the earthquake and tsunami, and (2) from the nuclear crisis. Total scores were calculated for the analyses (Cronbach's *α* = .95 for both).

### Demographic measures

Demographic measures included age, gender, area of residence, number of cohabitants, health conditions, alcohol and cigarette usage, and anamnestic history.

### Description of the disaster experience

Descriptions of disaster experience used in Dougall et al. [45] were modified to describe the experience of encountering the earthquake and tsunami using 23 items measured in yes/no responses. If the responses were yes to a particular experience, such as financial loss, then an open-answer question followed to allow participants to freely describe their experience of financial loss. The nuclear crisis was measured using 14 items in the same way. For instance, open-answer questions included financial loss, personal loss, and a description of the encounter. Descriptive statistics for the main parts of the obtained information are presented in Table S1.

### WHOQOL-BREF

The twenty-six item WHOQOL-BREF in Japanese has been widely used to measure quality of life, which is defined as the personal evaluation of one's life condition based on one's own goals, expectations, and standards including subscales such as physical QoL, psychological QoL, social relationships, and environment in 5-point Likert-type graded responses (1 = not at all and 5 = extremely/completely). The scale was originally developed and validated by the WHO [46], and the Japanese translation, back translation and validation were conducted by Tazaki & Nakane [47]. Scores were calculated as the means of 26 items (Cronbach's *α* = .93).

### Perceived Stress Scale (PSS)

The fourteen item PSS has been widely used to measure general stress in 5-point Likert-type graded responses (0 = never and 4 = very often). The scale was originally developed and validated by Cohen et al. [48], and the Japanese translation, back translation and validation for the Japanese perceived stress scale (JPSS) were conducted by Iwahashi et al. [49]. Total scores were used for the analyses (Cronbach's *α* = .85).

### Jichi Medical School Social Support Scale (JMS-SSS)

The fourteen item JMS-SSS, which was developed and validated by Tsutsumi et al. [50], has been widely used in Japan to measure social support in 4-point Likert-type graded responses (1 = strongly agree and 4 = do not agree at all). Total scores were used for the analyses (Cronbach's *α* = .92).

### Posttraumatic Growth Inventory (PTGI)

The twenty-one item PTGI has been widely used to measure trauma-related growth including subscales such as relationships with others, new possibilities, strength, and spirituality in 6-point Likert-type graded responses (0 = did not experience and 5 = to a very great degree). The scale was originally developed and validated by Tedeschi & Calhoun [51], and the Japanese translation and validation was conducted by Taku et al., [52]. Total scores were used for the analyses (Cronbach's *α* = .76).

### Cognitive Appraisal of Health Scale (CAHS)

The twenty-eight item CAHS has been widely used to measure health-related appraisal including subscales such as harm/loss (8 items), benign/irrelevant, threat, and challenge in 5-point Likert-type graded responses (1 = strongly disagree and 5 = strongly agree). The scale was originally developed and validated by Kessler [53], and the Japanese translation and back translation were conducted by the authors for the current study. Internal consistency (Cronbach's *α*) for harm/loss, benign/irrelevant, threat, and challenge subscales were .89, .66, .76, and .79, respectively. Total scores for each subscale were used for the analyses.

### Hospital Anxiety and Depression Scale (HADS)

The fourteen item HADS has been widely used to measure self-reported symptoms for anxiety (7 items) and depression (7 items) in 5-point Likert-type graded responses (1 = strongly disagree and 5 = strongly agree). The scale was originally developed and validated by Zigmond & Snaith [54], and the Japanese translation and back translation were conducted by Hatta et al. [55]. Internal consistency (Cronbach's *α*) for anxiety and depression subscales were .84 and .79, respectively. Total scores for each subscale were used for the analyses.

### Data analyses

Pre-analyses were conducted before the main analyses. First, a within-participants t-test was conducted to determine whether the degree of PTS from the earthquake and tsunami differed from that of the nuclear crisis. Then, the effect of area was examined using a one-way between-participant ANOVA on PTS. Finally, correlational analyses were conducted among variables in the hypothesized models to exclude unnecessary paths (*r*<.20) from the models.

A path model using the SEM procedure with maximum-likelihood estimation was conducted to evaluate the hypothesized models in which goodness of fit between restructured correlations based on the hypothesized model and observed correlations were examined. Model-trimming and model-building approaches were taken; hypothesized paths with low standardized coefficients (<.15) were eliminated, and a modification index estimated by AMOS as larger than 50 was added as a path. The models were evaluated by diagnostic parameters such as Chi-square for the hypothesized model (*χ^2^_model_*), goodness-of-fit index (GFI>.9), the comparative-fit-index (CFI>.90), and the standardized root mean square residual (SMR<.08) based on conventional criteria [56] [57] [58] [59]. Comparison among models was based on Akaike information criterion (AIC), which is often used for comparison against non-nested models [56] [60] [61]. Although absolute value of the AIC is not intuitively meaningful by itself, a smaller value indicates more a parsimonious and more informative model in comparison with other models.

Lastly, the effect of biases in the Internet survey was explored. Differences in basic demographic and socioeconomic factors between the national population and the sample in the current study were examined for age, gender, achieved level of education, and income level using the *χ^2^* test.

### Ethics statement

The institutional ethical committee at Jichi Medical University approved all materials and protocols of the study.

## Results

### Pre-analyses

Characteristics of the participants are presented in Table S1. Range, mean, and SD for each scale and subscale are shown in Table 1. In comparison of PTS type, IES-R for the earthquake and tsunami was substantially higher than that for the nuclear crisis, *t* (3487) = 20.4, *p*<.001, *η_p_^2^* = .11. Because the current study centers on PTS from the earthquake and tsunami, that is where the subsequent modeling focuses. In addition, IES-R for the earthquake and tsunami was substantially higher among participants from the primary disaster area, *F* (1, 3485) = 147.5, *p*<.001, *η_p_^2^* = .08. Correlation coefficients among variables are shown in Table 2. Demographic variables were not strongly associated with IES-R for the earthquake and tsunami. Because the correlation between harm/loss and threat subscales in the CAHS was substantially high, a composite variable was constructed. The remaining hypothesized paths were examined using path models since they were above the criteria (*r*>.2).

**Table 1:** Descriptive statistics of the relevant scales.

Scale	Subscale	Range	Mean±SD
**CAHS**			
	Threat	5.00–25.00	13.91±3.92 (14.87±3.68)
	Harm/loss	8.00–40.00	18.97±6.83 (22.82±6.06)
	Benign	4.00–20.00	10.33±3.11 (9.19±2.79)
	Challenge	6.00–30.00	18.55±4.31 (19.99±4.00)
	Negative	1.31–65.00 (13.00±64.00)	32.87±9.88 (37.69±9.04)
**IES-R for the earthquake & tsunami**		0.00–61.00	11.95±12.94 (17.01±13.36)
	Intrusion	0.00–30.00	4.21±4.84 (6.27±5.73)
	Avoidance	0.00–27.00 (0.00–25.00)	4.21±4.87 (5.62±5.09)
	Hyperarousal	0.00–20.00	3.46±3.93 (5.12±4.14)
**IES-R for the nuclear crisis**		0.00–61.00	9.14±12.45 (11.74±13.20)
	Intrusion	0.00–30.00	3.52±5.25 (4.60±5.84)
	Avoidance	0.00–30.00 (0.00–26.00)	2.91±4.52 (3.54±4.77)
	Hyperarousal	0.00–22.00 (0.00–21.00)	2.70±3.79 (3.61±4.03)
**JPSS**		0.00–56.00 (7.00–56.00)	27.48±8.05 (28.94±7.83)
**PTGI**		21.00–126.00	48.12±22.06 (54.43±21.90)
	Relationship	6.00–36.00	14.24±6.89 (16.04±6.93)
	New possibility	4.00–24.00	8.60±4.41 (9.37±4.54)
	Strength	4.00–24.00	8.90±4.64 (10.16±4.77)
	Spirituality	4.00–24.00	9.24±4.36 (10.09±4.38)
**HADS**			
	Anxiety	7.00–28.00	14.89±3.79 (15.58±3.65)
	Depression	7.00–28.00	17.23±3.57 (17.21±3.29)
**WHOQOL-BREF** 1		1.08–5.00 (1.15–5.00)	3.09±.57 (2.98±.53)
	Physical function	1.00–5.00 (1.14–5.00)	3.32±.63 (3.22±.63)
	Mental function	1.00–5.00	3.01±.71 (2.92±.65)
	Social function	1.00–5.00	2.98±.74 (2.91±.72)
	Environment	1.00–5.00	3.01±.71 (2.89±.59)

Numbers shown in parentheses are for participants from the primarily devastated areas.

1 Item averages were calculated for the WHOQOL-BREF total score and the subscale scores.

**Table 2:** Correlation among variables in the hypothesized models.

	1.	2.	3.	4.	5.	6.	7.	8.	9.	10.
**1. Harm/Loss (CAHS)**	–	**.71** ***	**−.34** ***	−.02	**.41** ***	**.36** ***	.14***	**.42** ***	.19***	**−.39** ***
**2. Threat (CAHS)**		–	**−.36** ***	−.11***	**.35** ***	**.42** ***	.04	**.50** ***	.18***	**−.40** ***
**3. Benign (CAHS)**			–	.04	−.16***	**−.21** ***	−.04	**−.26** ***	−.02	.17***
**4. Challenge (CAHS)**				–	.00	**−.34** ***	**.42** ***	−.04	**−.35** ***	**.39** ***
**5. IES-R**					–	**.26** ***	**.31** ***	**.45** ***	.12**	**−.31** ***
**6. JPSS**						–	.13***	**.37** ***	**.31** ***	**−.62** ***
**7. PTGI**							–	.17***	−.18***	**.21** ***
**8. Anxiety (HADS)**								–	−.06	**−.32** ***
**9. Depression (HADS)**									–	**−.44** ***
**10. WHOQOL-BREF**										–

Results of correlation analyses among participants from the primary disaster area are shown (*n*  =  1083). Correlation coefficients above .20 are in bold.

**, *p* < 0.01;

***, *p* < 0.001.

### Comparison among the hypothesized models

**Figure 3:**
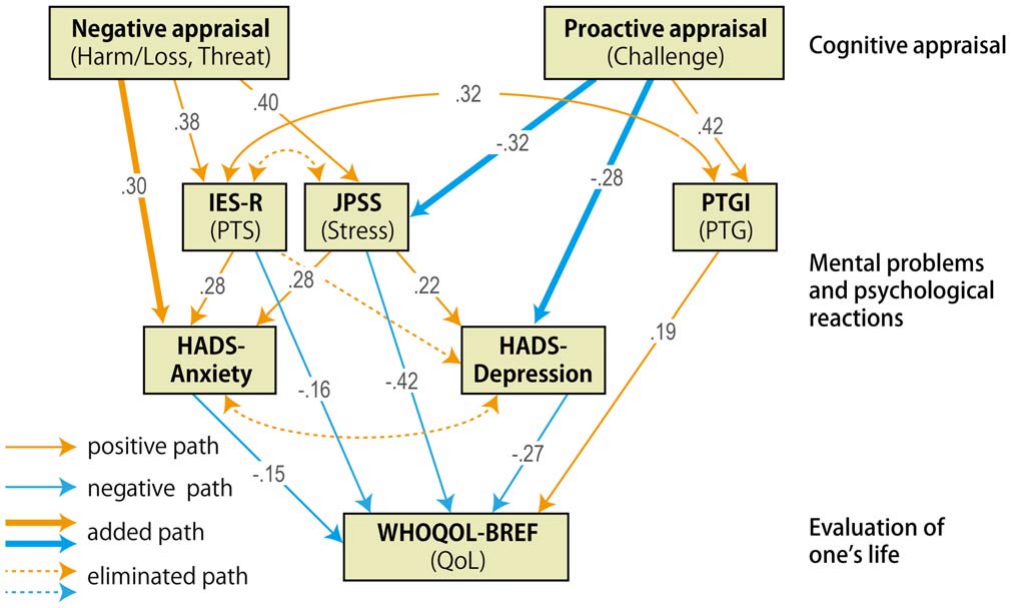
Model 1 with QoL as a final outcome after modifications. This model exhibited a higher and more sufficient goodness of fit, and thus may better represent the psychological adaptation process after the earthquake. One-headed arrows indicate the direction of observed regression. Two-headed arrows indicate observed correlation, with error components being omitted for simplicity. Additional observed paths are indicated as bold-lined arrows, and unsupported paths are shown as dotted-lined arrows. The numbers on the arrows represent regression coefficients.

**Figure 4:**
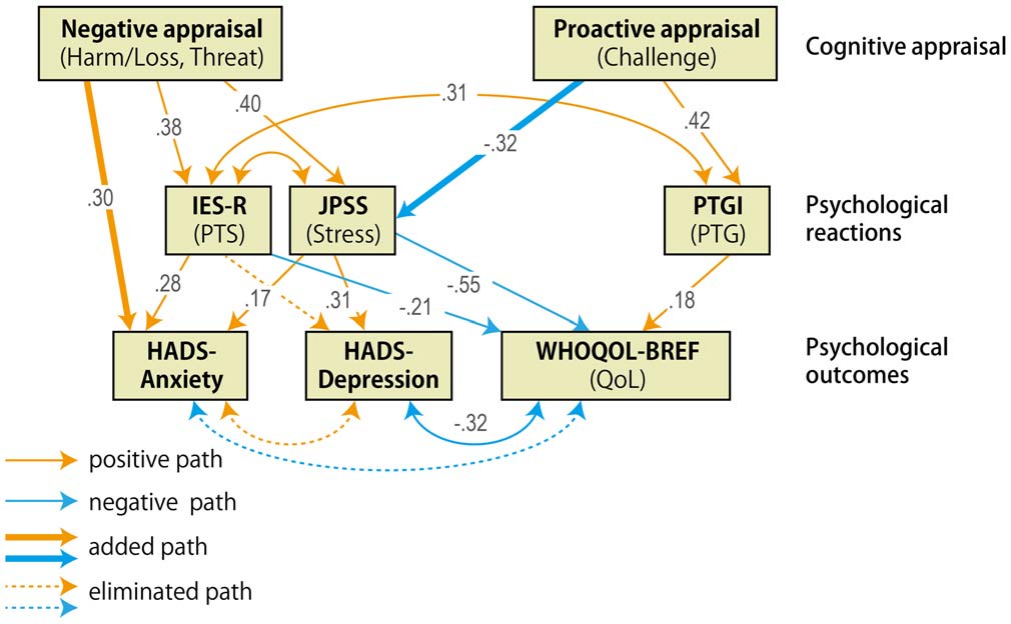
Model 2 with QoL and mental problems as final outcomes. This model has only marginal goodness of fit, and thus is less preferable than Model 1. One-headed arrows indicate the direction of observed regression. Two-headed arrows indicate observed correlation, with error components being omitted for simplicity. Additional observed paths are indicated as bold-lined arrows, and unsupported paths are shown as dotted-lined arrows. The numbers on the arrows represent regression coefficients.

Two path models (Figures 1 and 2) were examined. Goodness of fit indices improved after the posthoc modifications based on the criteria discussed in data analyses for all the hypothesized models. Model 1 (Figure 3) showed a sufficient fit and Model 2 (Figure 4) showed a marginal fit (Table 3). Standardized path coefficients are shown in Figures 3 and 4 respectively. In a comparison between the models after the modifications based on the AIC, Model 1 reflected observed relationships in a more parsimonious and informative way than it did with the other model.

**Table 3:** Goodness of fit indices for the baseline (hypothesized) and modified models.

	df	GFI	CFI	RMR	AIC
Model 1					
Hypothesized	14	.91	.80	.12	545.56
Modified	13	.96	.92	.06	248.83
Model 2					
Hypothesized	14	.92	.82	.12	490.13
Modified	13	.93	.88	.08	360.71

Modification was performed based on the pre-analyses and the results of the baseline models. All the paths retained for each model were significant at (*p* < .001).

Model 1 with the better fit was also applied to participants from the other two areas. However, the goodness of fit for the modified version was not adequate (GFI = .92, CFI = .83, standardized RMR = .11, AIC = 947.1.

### Effects of Internet survey

To assess possible biases of an Internet-based survey, we examined differences in demographics and socioeconomic statuses between the national population and the sample in the current study (see Appendix S1 for detailed data). Significant differences were found for age (5 age categories: 20s to 60s): *χ^2^* (*n* = 3455, *df* = 4) = 87.84, *p*<.001; gender (female/male): *χ^2^* (*n* = 3455, *df* = 1) = 92.06, *p*<.001; achieved level of education (high school, 2 years of college, 4 years of college or higher): *χ^2^* (*n* = 3439, *df* = 2) = 2870.09, *p*<.001; and income level (eight levels), *χ^2^* (*n* = 2977, *df* = 7) = 2870.09, *p*<.001. In short, the samples in the current study were older, included more males, had attained higher educations, and were more concentrated in an annual household income below ¥ 4 million. However, none of these demographic and socioeconomic factors were associated with PTS and PTG in the current study.

## Discussion

The current study aimed to comprehensively model early psychological adjustment to an earthquake three months after its occurrence. Based on past studies, we hypothesized two models (QoL as a final outcome vs. QoL and mental problems together as outcomes) with a central focus on PTS. The most fitted path model was achieved for the model with QoL as a final outcome in the most directly exposed participants (modified version of Model 1).

### General findings

In the process of building this model, the following general findings were observed. First, in a comparison between the PTS from the natural disaster with that from the artificial disaster, PTS from the earthquake and tsunami was substantially greater than that from the nuclear crisis, which contradicts Galea, Nandi, & Vlahove [62] where an artificial disaster resulted in higher PTS. On the other hand, our finding is consistent with reviews [2] [14] [15] that reported that natural disasters have greatest impact at first while technological disaster have a greater propensity to evoke chronic stress. This finding led us to focus on the effects of the earthquake and tsunami rather than on those of the nuclear power plant accidents.

Second, PTS from the natural disaster was higher in the primarily affected area, which is similar to previous findings [40]. This suggests that the rescue and care after such a large natural disaster should be intensively and promptly allocated to the people directly affected due to the substantially high PTS among them.

Third, demographic effects such as age and gender on PTS were not supported in the current study. Null demographic effects are not rare in the case of an extremely severe traumatic event where many people are affected irrelevant of their demographic background [20]. This phenomenon may well reflect the magnitude and severity of this disaster.

In addition, social support did not alleviate PTS, which contradicts Wang et al. [40]. However, we cannot deny that the effect of demographics and social support might be effective at a later stage of psychological adjustment.

### Interpretation and implication of the model

Among the hypothesized models, Model 1 (QoL as a final outcome), most likely reflects what was cognitively and psychologically going on among people in the primarily affected area at three months after the earthquake. However, model fit for the modified version of Model 1 was not adequate for participants from other areas, possibly due to the restricted range problem [63] for negative psychological constructs such as PTS.

Cognitive appraisal appears to have preceded and accounted for other psychological reactions to the 2011 Tohoku earthquake and tsunami among people in primarily devastated areas. Because cognitive appraisal is theorized as an acquirable skill rather than an innate trait, interventions could be tailored to help victims appraise a situation in a more proactive manner and identify and challenge negative trauma-related cognitions. For example, cognitive restructuring has been used successfully as a therapeutic technique for decreasing maladaptive cognitions associated with trauma, including in the treatment of PTSD [64]. Other cognitive behavioral therapy techniques have been useful in treating symptoms of posttraumatic stress by reducing negative emotions associated with cognitive appraisals of the event and by creating memory associations that compete with the retrieval of negative memories [65]. Such intervention would require a relatively small number of training sessions and relatively few resources [66]. In addition, cognitive intervention could be incorporated into emergency drills as preventative training for cognition. Researchers are currently investigating the effectiveness of prophylactic military training interventions to reduce or eliminate combat-related stress reactions [67]. If successful, these interventions could be modified and disseminated to other populations who are at risk for trauma exposure.

PTS accounted for anxiety and QoL, as with previous findings [37] [68]. However, PTS does not appear to have a devastating influence on other psychological sequelae compared with general stress at three months after the earthquake and tsunami. In particular, general stress, not PTS, accounted for the degree of depression post-disaster. Also, the relatively low correlation between PTS and PTG indicates that PTS may not be a prerequisite for PTG. Rather, PTS likely precedes correlates of PTG as Zollner et al. [21] proposed.

As opposed to PTS, PTG appears to have a positive effect on QoL, but it does not have an alleviating effect on general stress, depression, and anxiety at this point. The alleviating effect of PTG is likely dependent on the type, quality, and magnitude of a trauma because the association between PTG and other psychological sequelae tends to be inconsistent [21]. For instance, some studies have reported that PTG alleviates suicide ideation and improves quality of life [69], [70] while others have reported no actual positive effects [21]. In the current study, PTG did not reduce the negative psychological sequelae at this acute stage. Because PTG has been conceptualized as a process that unfolds over time as victims recover from their traumatic experiences, it may be that earlier reports of PTG represent deliberate coping efforts evoked by distress and aimed at alleviating symptoms [71], [72]. However, PTG is likely to predict reductions in psychological sequelae after more time passes, as has been demonstrated following exposure to other types of disaster [73].

In short, the paths to depression (from negative cognitive appraisal via general stress) and to anxiety (from challenge appraisal via PTS) are two distinct routes, suggesting that early intervention for depression and anxiety should be distinguished accordingly. As for the paths to QoL, the effect of negative routes (from negative cognitive appraisal, PTS, and general stress) was greater than that of positive routes (from positive cognitive appraisal and PTG). This suggests that reducing negative cognitive appraisal would be more useful in preventing the degradation of QoL via PTS and general stress than would fostering positive appraisal at an early phase of psychological adjustment.

### Limitations

There are several limitations that should be noted. First, because the model was developed based on previous studies and established theories, it was mainly tested in a confirmatory manner. However, this may be an unavoidable limitation for studies on disasters as they are basically unpredictable. For further validation, the psychological constructs and models that were measured concurrently should also be tested in a longitudinal manner.

Second, the degree of exposure to the disaster was not objectively controlled except for by location at the time of the earthquake. This factor would preferably be incorporated into the model, but is difficult to collect in Internet surveys. Although Internet surveys allow the acquisition of a sufficient number of samples to generate holistic models, detailed examination fine-tuned for personal experience of the disaster would be better evaluated through traditional, interview-based designs with biological measurements.

Third, the Internet sample might not be representative of the population. However, despite the common belief that there are large biases in Internet samples, they are found to be similar to the general population with the exception of a few demographic factors such as age, gender, level of education, and urbanity of residence [74]. In the current study, rather than avoiding such possible Internet biases, we chose to describe actual biases in the Internet survey and assess whether such differences may have affected the findings of the current survey. As predicted based on Ross et al [74], we found biases in age, gender, and level of education. In addition, the low-income group was inflated, probably because members of this socioeconomic group may be more attracted to the rewards of Internet surveys. Although there were apparent differences in demographic and socioeconomic factors between the general population and the sample of the current study, none of these factors were significantly associated with PTS and PTG. Thus, we can expect that the biases in the Internet survey did not affect the major findings of the current study. In addition, given the high Internet literacy with a penetration rate of 78% in Japan [75] and the fact that 99% of the Internet accounts of major corporations were restored within two months of the earthquake [76], the Internet survey may well reflect the psychological state of the majority of Japanese.

However, this does not necessarily mean we can neglect the presence of Internet minorities. Since they tend to be aged adults in rural areas, their suffering may be qualitatively different from that of people in the surveyed population. Here again, interview-based surveys may be supplemented to achieve a genuinely holistic picture of the psychological adaptation process for the earthquake reflecting the whole population.

### Conclusion

In conclusion, the current study successfully modeled the structure of the psychological adjustment to an earthquake, depicting paths coming from both positive and negative cognitive appraisals of the disaster, relating to different facets of mental disorders in the form of general stress, anxiety, depression, PTS with detectable signs of PTG, and finally leading to perceived QoL as a final outcome. To the best of our knowledge, this is the first comprehensive psychological model that sufficiently depicts the psychological adaptation process of a general population to large-scale natural disasters.

The model suggests the importance of clinical intervention to reduce negative cognitive appraisal, and for caring for general stress and PTS to maintain QoL at an early stage of psychological adaptation to a disaster. Albeit smaller, the model also depicted the presence of a positive route via which positive cognitive appraisal facilitates PTG to achieve a higher QoL, suggesting the potential importance of positive psychological preventive care in preparation for unexpected natural disasters.

Since the current study focused on a cross-sectional analysis of the early psychological adaptation process, whether time will resolve the general stress and PTS, and possibly foster PTG remains uncertain. Longitudinal studies that are currently underway will offer clarity to this issue.

Finally, we hope the knowledge that we have drawn from analyzing the psychological adaptation processes of the direct and indirect victims of the Tohoku earthquake, tsunami, and nuclear power plant accident will serve as a valuable learning tool to help understand the psychological adaptation process of potential victims of the unpredictable disasters that inevitably happen throughout the world.

## Supporting Information

Table S1 **Characteristics of samples.**
(DOC)

Appendix S1 **Differences in basic demographic and socioeconomic factors between the national population and the sample in the current study.**
(DOC)

## References

[1] U. S. Geological Survey (2011). Magnitude 9.0 - near the east coast of Honsyu, Japan.. http://earthquake.usgs.gov/earthquakes/eqinthenews/2011/usc0001xgp/#summary.

[2] Norrris FH, Friedman MJ, Watson PJ, Byrne CM, Diaz E (2002). 60,000 Disaster victims speak: Part I. An empirical review of the empirical literature, 1981–2001.. Psychiatry.

[3] Kutz I, Dekel R (2006). Follow-up of victims of one terrorist attack in Israel: ASD, PTSD and the perceived threat of Iraqi missile attacks.. Pers Indiv Differ.

[4] Nemeroff CB, Bremner JD, Foa EB, Mayberg HS, North CS (2006). Posttraumatic stress disorder: a state-of-the-science review.. J Psychiatr Res.

[5] Ford E, Ayers S, Bradley R (2010). Exploration of a cognitive model to predict post-traumatic stress symptoms following childbirth.. J Anxiety Disord.

[6] Karatzias T, Chouliara Z (2009). Cognitive appraisals and physical health in people with posttraumatic stress disorder (PTSD).. Med Hypotheses.

[7] Cao H, Alxander C, Mcfarlane S (2003). Prevalence of psychiatric disorder following the 1988 Yun Nan (China) earthquake.. Soc Psychiatry Psychiatr Epidemiol.

[8] Chen C, Lin S, Tang H, Shen W, Lu M (2001). The Chinese version of the Davidson Trauma Scale: a practice test for validation.. Psychiatry Clin Neurosci.

[9] Goto T, Wilson JP (2003). A review of the history of traumatic stress studies in Japan: from traumatic neurosis to PTSD.. Trauma Violence Abuse.

[10] Kokai M, Fujii S, Shinfuku N, Edwards G (2004). Natural disaster and mental health in Asia.. Psychiaty Clin Neurosci.

[11] Liu A, Tan H, Zhou J, Li S, Yang T (2006). An epidemiologic study of posttraumatic stress disorder in flood victims in Hunan China.. Can J Psychiatry.

[12] Kun P, Chen X, Han S, Gong X, Chen M (2009). Prevalence of post-traumatic stress disorder in Sichuan Province, China after the 2008 Wenchuan earthquake.. Public Health.

[13] Zlotnick C, Franklin CL, Zimmerman M (2002). Does subthreshold posttraumatic stress disorder have any clinical relevance?. Compr Psychiatry.

[14] Norris F, Friedman J, Watson P (2002). 60,000 Disaster victims speak: part II. summary and implicatoin s of the disaster mental health research.. Psychiatry.

[15] Baum A, Frederick CJ, Frieze Ih, Shneidman ES, Wortman CB (1987). Cataclysms, crises, and catastrophes: Psychology in action.

[16] Galea S, Ahern J, Resnick H, Kilpatrick D, Bucuvalas M (2002). Psychological sequelae of the September 11 terrorist attacks in New York City.. N Eng J Med.

[17] Tedeschi RG, Calhoun LG, Cann A (2007). Evaluating resource gain: understanding and misunderstanding posttraumatic growth.. Appl Psychol.

[18] Sumalla EC, Ochoa C, Blanco I (2009). Posttraumatic growth in cancer: reality or illusion?. Clin Psychol Rev.

[19] Rajandram RK, Jenewein J, McGrath CPJ, Zwahlen RA (2010). Posttraumatic growth: a novel concept in oral cavity cancer care?. Oral Oncol.

[20] Dekel R, Nuttman-Shwartz O (2009). Posttraumatic stress and growth: the contribution of cognitive appraisal and sense of belonging to the country.. Health Soc Work.

[21] Zoellner T, Maercker A (2006). Posttraumatic growth in clinical psychology — a critical review and introduction of a two component model.. Clin Psychol Rev.

[22] Nishi D, Matsuoka Y, Kim Y (2010). Posttraumatic growth, posttraumatic stress disorder and resilience of motor vehicle accident survivors.. Biopsychosoc Med.

[23] Stanton A, Bower J, Low C, Calhoun LG, Tedeschi RG (2006). Posttraumatic growth after cancer.. Handbook of posttraumatic growth: research and practice.

[24] Xu J, Liao Q (2011). Prevalence and predictors of posttraumatic growth among adult survivors one year following 2008 Sichuan earthquake.. J Affect Disord.

[25] Lazarus RS, DeLongis A, Folkman S, Gruen R (1985). Stress and adaptational outcomes: the problem of confounded measures.. Am Psychol.

[26] Dysvik E, Natvig GK, Eikeland OJ, Lindstrøm TC (2005). Coping with chronic pain.. Int J Nurs Stud.

[27] Joksimovic L, Starke D, v d Knesebeck O, Siegrist J (2002). Perceived work stress, overcommitment, and self-reported musculoskeletal pain: a cross-sectional investigation.. Int J Behav Med.

[28] Jones DA, Rollman GB, White KP, Hill ML, Brooke RI (2003). The relationship between cognitive appraisal, affect, and catastrophizing in patients with chronic pain.. J Pain.

[29] Unruh AR, OT. Judith RN, Merskey HDM (1999). Does gender affect appraisal of pain and pain coping strategies?. Clin J Pain.

[30] Vlaeyen J, Linton S (2000). Fear-avoidance and its consequences in chronic musculoskeletal pain: a state of the art.. Pain.

[31] Ehlers A, Steil R (1995). Maintenance of intrusive memories in posttraumatic stress disorder: a cognitive approach.. Behav Cogn Psychother.

[32] Norberg MM, Diefenbach GJ, Tolin DF (2008). Quality of life and anxiety and depressive disorder comorbidity.. J Anxiety Disord.

[33] Johansen VA, Wahl AK, Eilertsen D, Weisaeth L, Hanestad BR (2007). The predictive value of post-traumatic stress disorder symptoms for quality of life: a longitudinal study of physically injured victims of non-domestic violence.. Health Qual Life Outcomes.

[34] Tsai K, Chou P, Chou F, Su T, Lin S (2007). Three-year follow-up study of the relationship between posttraumatic stress symptoms and quality of life among earthquake survivors in Yu-Chi, Taiwan.. J Psychiatr Res.

[35] Guan B, Deng Y, Cohen P, Chen H (2011). Relative impact of Axis I mental disorders on quality of life among adults in the community.. J Affect Disord.

[36] Noble AJ, Baisch S, Schenk T, Mendelow AD, Allen L (2008). Posttraumatic stress disorder explains reduced quality of life in subarachnoid hemorrhage patients in both the short and long term.. Neurosurgery.

[37] Morrill EF, Brewer NT, O'Neill SC, Lillie SE, Dees EC (2008). The interaction of post-traumatic growth and post-traumatic stress symptoms in predicting depressive symptoms and quality of life.. Psychooncology.

[38] Kocalevent RD, Levenstein S, Fliege H, Schmid G, Hinz A (2007). Contribution to the construct validity of the Perceived Stress Questionnaire from a population-based survey.. J Psychosom Res.

[39] Joseph S, Wood A (2010). Assessment of positive functioning in clinical psychology: Theoretical and practical issues.. Clin Psychol Rev.

[40] Wang X, Gao L, Zhang H, Zhao C, Shen Y (2000). Post-earthquake quality of life and psychological well-being: Longitudinal evaluation in a rural community sample in northern China.. Psychiatry Clin Neurosci.

[41] Xie XF, Wang M, Zhang RG, Li J, Yu QY (2011). The role of emotions in risk communication.. Risk Anal.

[42] Sutherland HJ, Lockwood GA, Tritchler DL, Sem F, Brooks L (1991). Communicating probabilistic information to cancer patients: is there ‘noise’ on the line?. Soc Sci Med.

[43] Horrowitz M, Wilner N, Alvarez W (1979). Impact of Event Scale: a measure of subjective stress.. Psychosom Med.

[44] Asukai N, Kato H, Kawamura N, Kim Y, Yamamoto K (2002). Reliability and validity of the Japanese-language version of the impact of event scale-revised (IES-R-J): four studies of different traumatic events.. J Nerv Ment Dis.

[45] Dougall A, Hayward M, Baum A (2005). Media exposure to bioterrorism: stress and the anthrax attacks.. Psychiatry.

[46] World Health Organization (1993). WHOQoL Study Protocol.

[47] Tazaki M, Nakane M (1997). WHOQOL 26 Manual Revised.

[48] Cohen S, Kamarck T, Mermelstein R (1983). A global measure of perceived stress.. J Health Soc Behav.

[49] Iwahasi S, Tanaka Y, Fukudo S, Hongo M (2002). The development of the Japanese version for the Perceived Stress Scale.. Jpn J Pyschosom Med.

[50] Tsutsumi A, Tsutsumi K, Kayaba K, Igarashi M (1998). Health-related behaviors, social support, and community morale.. Int J Behav Med.

[51] Tedeschi RG, Calhoun L (1996). The posttraumatic growth inventory: measuring the positive legacy of trauma.. J Trauma Stress.

[52] Taku K, Calhoun LG, Tedeschi RG, Gil-Rivas V, Kilmer RP (2007). Examining posttraumatic growth among Japanese university students.. Anxiety Stress Coping.

[53] Kessler TA (1998). The Cognitive Appraisal of Health Scale: development and psychometric evaluation.. Res Nurs Health.

[54] Zigmond AS, Snaith RP (1993). Hospital anxiety and depression scale.. Acta Psychitr Scand.

[55] Hatta H, Higashi A, Yashiro H, Ozasa K, Hayashi K (1998). A validatoin of the Hospital Anxiety and Depression Scale.. Jpn J Psychosom Med.

[56] Tabachnik B, Fidell L (2007). Using multivariate statistics.

[57] Mertler C, Vanatta R (2008). Advanced and multivariate statistical methods (3rd ed.).

[58] Bentler P (1988). Comparative fit indexes in structural models.. Psychol Bull.

[59] Tanaka J, Huba GJ (1989). A general coefficient of determination for covariance structure models under arbitrary GLS estimation.. Br J Math Stat Psychol.

[60] Akaike H (1987). Factor analysis and AIC.. Psychometrika.

[61] Bozdogen H (1987). Model selection and Akaike's information criteria (AIC): the general theory and its analytical extensions.. Psychometrika.

[62] Galea S, Nandi A, Vlahov D (2005). The epidemiology of post-traumatic stress disorder after disasters.. Epidemiol Rev.

[63] Nunnally J, Bernstein IH (1994). Psychometric theory (3rd Ed.).

[64] Mueser KT, Rosenberg SD, Rosenberg HJ (2009). Treatment of posttraumatic stress disorder in special populations: a cognitive restructuring program.

[65] Brewin CR, Holmes EA (2003). Psychological theories of posttraumatic stress disorder.. Clin Psychol Rev.

[66] McAllister M, McKinnon J (2009). The importance of teaching and learning resilience in the health disciplines: a critical review of the literature.. Nurse Educ Today.

[67] Novotney A (2009). Strong in mind and body.. Monitor Psychol.

[68] Thompson KE, Vasterling JJ, Benotsch EG, Brailey K, Constans J (2004). Early symptom predictors of chronic distress in Gulf War veterans.. J Nerv Ment Dis.

[69] Cann A, Calhoun LG, Tedeschi RG, Solomon DT (2010). Posttraumatic growth and depreciation as independent experiences and predictors of well-being.. J Loss Trauma.

[70] Yu XN, Lau JTF, Zhang J, Mak WWS, Choi KC (2010). Posttraumatic growth and reduced suicidal ideation among adolescents at month 1 after the Sichuan Earthquake.. J Affect Disord.

[71] Calhoun LG, Tedeschi RG, Calhoun LG, Tedeschi RG (2006). The foundations of posttraumatic growth: an expanded framework.. Handbook of posttraumatic growth: research and practice.

[72] Park CL (2010). Making sense of the meaning literature: An integrative review of meaning making its effects on adjustment to stressful life events.. Psychol Bull.

[73] McMillen JC, Smith EM, Fisher RH (1997). Perceived benefit and mental health after three types of disaster.. J Consult Clin Psychol.

[74] Ross MW, Månsson SA, Daneback K, Cooper A, Tikkanen R (2005). Biases in internet sexual health samples: comparison of an internet sexuality survey and a national sexual health survey in Sweden.. Soc Sci Med.

[75] Ministry of Internal Affairs and Communication (2011). White paper 2011, Information and Communication in Japan.. http://www.soumu.go.jp/johotsusintokei/whitepaper/eng/WP2011/2011-index.html.

[76] Setoyama J (2011). Higashinihon daishinsai ni okeru jyohotsushin bunya no omona torikumi (Major efforts of information and communication fields regarding the Great East Japan Earthquake).. Rippo to Chosa (legislation and investigation).

