# Supplementary material for: Cognitive and Psychological Reactions of the General Population Three Months After the 2011 Tohoku Earthquake and Tsunami
Source: PLoS One. 2012 Feb 8;7(2):e31014. doi: 10.1371/journal.pone.0031014 (PMC3275613; doi:10.1371/journal.pone.0031014)
Supplement: Table S1 — Characteristics of samples. (DOC) [file pone.0031014.s001.doc]

Table S1. Characteristics of samples

***n %***

**Demographics**

Marital status

Married 2353 (713) 68.1 (65.8)

Never married/Divorced 1043 (354) 30.2 (32.7)

Widowed 59 (16) 1.7 (1.5)

Education

Junior high school 72 (22) 2.1 (2.0)

High school 1028 (399) 29.8 (36.8)

Associate degree 750 (224) 21.7 (20.7)

College degree 1441 (407) 41.7 (37.6)

>College degree 148 (27) 4.3 (2.5)

N.A. 16 (4) .5 (.4)

Annual household income (in \)^1^

<3,990,000 922 (306) 26.7 (28.3)

4,000,000-4,990,000 488 (157) 14.1 (14.5)

5,000,000-5,990,000 382 (133) 11.1 (12.3)

6,000,000-6,990,000 292 (91) 8.5 (8.4)

7,000,000-7,990,000 251 (79) 7.3 (7.3)

8,000,000-8,990,000 184 (56) 5.3 (5.2)

9,000,000-9,990,000 178 (48) 5.2 (4.4)

10,000,000-11,990,000 152 (42) 4.4 (3.9)

12,000,000-14, 990,000 128 (28) 3.7 (2.6)

>15,000,000 71 (13) 2.1 (1.2)

N.A. 407 (130) 11.8 (12.0)

Employment

Full time 1918 (611) 55.5 (56.4)

Part time 379 (118) 10.9 (10.9)

Student 94 (26) 2.7 (2.4)

Unemployed 404 (130) 11.7 (12.0)

House wife/husband 599 (177) 17.3 (16.3)

Other/N.A. 61 (21) 1.9 (1.9)

**Self-reported medical history**

Prior illness

Yes 760 (261) 22.0 (24.1)

No 2695 (822) 78.0 (75.9)

Prior mental disorder

Yes 178 (63) 5.2 (5.8)

No 3276 (1019) 94.8 (94.2)

Prior PTSD

Yes 8 (4) .2 (.4)

No 2447 (1079) 99.8 (99.6)

**Experience of disaster**

Financial loss due to the disaster

Yes 1071 (757) 31.0 (69.9)

No 2384 (326) 69.0 (30.1)

Acquaintance affected

Yes 1346 (818) 39.0 (75.5)

No 2109 (265) 61.0 (24.5)

Injury due to the earthquake

Yes 78 (65) 2.3 (6.0)

No 3377 (1018) 97.7 (94.0)

Relocation due to the disaster

Yes 66 (53) 1.9 (4.9)

No 3389 (1030) 98.1 (95.1)

Any involvement in rescue

Yes 144 (109) 4.2 (10.1)

No 3311 (974) 95.8 (89.9)

Acquaintance involved in dealing

with the nuclear crisis

Yes 165 (86) 4.8 (7.9)

No 3290 (997) 95.2 (92.1)

Financial loss due to the nuclear crisis

Yes 319 (160) 9.2 (14.8)

No 3136 (923) 90.8 (85.2)

Move due to the nuclear crisis

Yes 43 (27) 1.2 (2.5)

No 3442 (1056) 98.8 (97.5)

Numbers shown in parentheses are for participants from the primarily devastated areas. “*N.A.*” is the not-answered item. ^1^Monthly currency exchange rate for June of 2011 was $1.00 = \80.52.
