# Supplementary material for: Cognitive and Psychological Reactions of the General Population Three Months After the 2011 Tohoku Earthquake and Tsunami
Source: PLoS One. 2012 Feb 8;7(2):e31014. doi: 10.1371/journal.pone.0031014 (PMC3275613; doi:10.1371/journal.pone.0031014)
Supplement: Appendix S1 — Differences in basic demographic and socioeconomic factors between the national population and the sample in the current study. (DOC) [file pone.0031014.s002.doc]

**­­­­­­**Differences in basic demographic and socioeconomic factors between the national population and the sample in the current study were examined for age, gender, achieved level of education, and income level. Demographic and socioeconomic data for the general population were according to The Statistics Bureau and the Director-General for Policy Planning of Japan and statistical research and training institute, Ministry of internal affairs and communications (2010). Specific results are as indicated below.

1. **Age (5 age categories: 20s to 60s)**

There was significant difference in age distribution: *χ^2^* (*n* = 3455, *df* = 4) = 87.84, *p* < .001. In general, the sample in the current study was older than the general population based on the adjusted standardized residuals. Age was not associated with PTS or PTG in the current study, as mentioned in the main text (*r* (3455) = .02, *N.S.* for both).

Age difference between the national population and the sample in the current study

Note: Proportion = the number of observations in the cell above divided by the total number of observations for a column.

1. **Gender**

There was significant difference in gender distribution: *χ^2^* (*n* = 3455, *df* = 1) = 92.06, *p* < .001. The sample in the current study had a higher proportion of males than that of the general population based on national data. This is a typical bias according to Ross et al. (2005). Gender was not associated with PTS or PTG in the current study, as mentioned in the main text (*r* (3455) = .04, *N.S.* and .08, *N.S.*, respectively).

Gender difference between the national population and the sample in the current study

Note: Proportion = the number of observations in the cell above divided by the total number of observations for a column.

1. **Achieved level of education (high school, 2 years of college, 4 years of college or higher)**

There was significant difference in achieved level of education: *χ^2^* (*n* = 3439, *df* = 2) = 2870.09, *p* < .001. The sample in the current study had a higher education, which is a known bias for Internet samples according to Ross et al. (2005). Educational level was not associated with PTS or PTG in the current, study as mentioned in the main text (*r* (3439) = -.08, *N.S.*, and -.03, *N.S.*, respectively).

Difference in achieved level of education between the national population and the sample in the current study

Note: Proportion = the number of observations in the cell above divided by the total number of observations for a column.

1. **Income level (8 levels)**

There was significant difference in income level: *χ^2^* (*n*= 2977, *df* = 7) = 2870.09, *p* < .001. The sample in the current study was more concentrated at an annual household income of less than \4 million. Household income level was not associated with PTS or PTG in the current study, as mentioned in the main text (*r* (3439) = -.01, *N.S.* and .04, *N.S.*, respectively).

Difference in income level between the national population and the sample in the current study

Note: Proportion = the number of observations in the cell above divided by the total number of observations for a column. The lower limit of each quota is “more than or equal to” and the upper limit is “less than” (except for 4M and 10M, which are as indicated). M indicates million yen (\). Monthly currency exchange rate for June of 2011 was $1.00 = \80.52.

**References**

The Statistics Bureau and the Director-General for Policy Planning of Japan and statistical research and training institute, Ministry of internal affairs and communications. (2010). Todofuken shikutyoson no sugata (Outlook of prefectures and cities in Japan) (in Japanese). Available from <http://www.e-stat.go.jp/SG1/chiiki/Welcome.do?lang=01>. Accessed 2012, Jan 8.

Ross M W, Mansson S A, Daneback K, Cooper A, Tikkanen R. (2005). Biases in internet sexual health samples: Comparison of an internet sexuality survey and a national sexual health survey in Sweden. *Social Science & Medicine*, 61, 245–252.
